# From blood flow to organ function: The physiology of autoregulatory dynamics

**DOI:** 10.1113/EP092760

**Published:** 2025-04-09

**Authors:** Niels‐Henrik Holstein‐Rathlou, Donald J. Marsh

**Affiliations:** ^1^ Department of Biomedical Sciences University of Copenhagen Copenhagen Denmark; ^2^ Department of Medical Sciences Brown University Providence Rhode Island USA

Arterial blood pressure is not constant but varies at many frequencies (Holstein‐Rathlou et al., 1995; Marsh et al., 1990; Wagner & Persson, 1994). Part of this variability is due to diurnal rhythms, but significant fluctuations also occur at higher frequencies (Holstein‐Rathlou et al., 1995). These faster pressure fluctuations arise from multiple sources, including variations in autonomic nerve activity and physical activity. Such fluctuations pose a potential challenge to organs and tissues by causing variations in blood perfusion unrelated to the functional needs of the organs. However, most tissues and organs exhibit autoregulation of blood flow, a function that adjusts haemodynamic resistance to maintain a nearly constant perfusion despite variations in arterial pressure (Johnson, 1986). Autoregulation of blood flow is particularly pronounced in the brain and the kidney, two organs whose function depend critically on stable blood perfusion. By preventing excessive fluctuations in organ blood flow, autoregulation enables optimal organ function.

The mechanisms underlying autoregulation have been the subject of extensive research over the years. Two mechanisms – metabolic and myogenic – have been proposed to explain autoregulation (Bayliss, 1902; Johnson, 1986). The metabolic mechanism focuses on the washout of vasoactive substances from the tissues. A sudden reduction in arterial pressure leads to a decreased perfusion rate and tissue hypoxia, resulting in the accumulation of vasodilatory substances that reduce haemodynamic resistance, thereby restoring normal perfusion (Johnson, 1986). As originally proposed by Bayliss (1902), the myogenic mechanism is intrinsic to the resistance vessels whereby an increase in intravascular pressure leads to vasoconstriction and an increased haemodynamic resistance. When blood pressure increases the tension in the vessel wall increases, this is sensed by mechanosensitive cation channels in smooth muscle cells in the vessel wall, particularly the transient receptor channels (TRP). This leads to membrane depolarization, calcium influx through voltage‐sensitive calcium channels, and activation of the contractile machinery in vascular smooth muscle cells (Solano et al., 2025).

Whereas these two mechanisms mediate autoregulation in most tissues, the kidney appears to be unique. Renal blood flow exceeds the metabolic demands of the organ by a large factor, and the oxygen extraction is low compared to other organs. Consequently, even a substantial drop in blood pressure does not result in renal hypoxia, making it unlikely that the metabolic mechanism plays a major role in renal autoregulation. Instead, the kidney relies on a unique intrinsic mechanism within individual nephrons – the tubuloglomerular feedback (TGF) mechanism (Holstein‐Rathlou & Marsh, 1994b; Johnson, 1986). TGF was first described in 1965 in a series of micro‐puncture experiments by Thurau and Schnermann where they showed that an increase in the NaCl concentration at the macula densa, a specialized plaque of cells located at the end of the thick ascending limb of the loop of Henle, elicited a vasoconstriction of the afferent arteriole (Thurau & Schnermann, 1965). An increase in glomerular filtration rate (GFR) due to elevated arterial pressure results in an increased delivery of NaCl to the macula densa. This triggers the release of ATP, which is converted, through hydrolysis by the extracellular ecto‐5′‐nucleotidase, to adenosine. Adenosine binds to A1 receptors on smooth muscle cells of the afferent arteriole, inducing vasoconstriction and a reduction of GFR (Carlström et al., 2015).

The relative roles of TGF and the myogenic mechanism have been the subject of numerous studies covering several decades, without the emergence of a clear consensus. Most studies have used a steady‐state approach, where the response of renal blood flow to step changes in perfusion pressure is recorded (Carlström et al., 2015). An alternative to the steady‐state approach is to examine the dynamics of the autoregulatory response, the idea being that the impact of the two mechanisms could be separated based on differences in their dynamics. The earliest attempts at characterizing the dynamics of renal autoregulation were made by Basar, Weiss and coworkers (Basar & Weiss, 1967, 1968, 1970; Başar, Ruedas et al., 1968, 1968). Despite identifying two distinct processes – one with a time constant of approximately 8–10 s and another with a time constant of 20–30 s – they concluded that only the myogenic mechanism accounted for renal autoregulation (Başar & Weiss, 1970).

Several experimental methods have been used to study dynamics of renal autoregulation. A common method has been the use of a mechanical pump to induce changes in renal perfusion pressure, either in vivo by connecting the pump to the aorta of an animal (Holstein‐Rathlou et al., 1991; Sakai, Hallman et al., 1986) or by using an isolated perfused kidney (Başar & Weiss, 1968; Başar, Ruedas et al., 1968, 1968). By using the pump, it is possible to induce step changes in perfusion pressure, sinusoidal pressure variations, or broadband fluctuations in blood pressure while recording instantaneous renal blood flow. As an alternative to the use of a pump, broad‐band fluctuations in arterial pressure have been induced by the induction of atrial fibrillation (Daniels et al., 1990; Sakai, Hallman et al., 1986) or by a suprarenal aortic clamp (Wang et al., 1999). Other studies have used spontaneously occurring arterial pressure fluctuations as the input to the kidney (Cupples et al., 1996; Pires et al., 2001).

The results of the experiments have been analysed either in the time domain or in the frequency domain. Studies using a step response have typically used a time domain approach where an exponential function or a differential equation has been fitted to the data (Daniels & Arendshorst, 1990; Just, 2007; van Beek et al., 2008). From these fits, time constants characterizing the dynamic response can be estimated. When renal input pressure is perturbed by either single‐frequency or broadband forcings, the results are typically analysed in the frequency domain using a transfer function (Holstein‐Rathlou & Marsh, 1994b):

(1) $$H\left(f\right)=\frac{S_{xy}\left(f\right)\bar{x}}{S_{xx}\left(f\right)\bar{y}}\;,$$

where *S_xy_* (*f*) is the cross spectrum between arterial blood pressure and renal blood flow, *S_xx_* (*f*) the auto spectrum of the arterial blood pressure, and $\bar{x}$ and $\bar{y}$ the mean values of arterial blood pressure and renal blood flow. The absolute value of the transfer function, |*H*(*f*)|, represents the effectiveness (gain) of autoregulation as a function of perturbation frequency (*f*). Conceptually, the gain is the ratio between the normalized amplitude of the variation in blood flow divided by the normalized amplitude of the variation in the arterial pressure. A gain of 1 indicates no autoregulation, whereas a value below 1 signifies flow attenuation relative to pressure variation, a hallmark of autoregulation. Often, gain is expressed in units of decibels (dB) (10 log_10_(|*H*(*f*)|), where negative values indicate autoregulation.

As mentioned above, data from dynamic studies of autoregulation have often been analysed, particularly in the time domain, by fitting various equations – such as differential equations or exponential functions – to the data. A key issue with this approach is that it assumes prior knowledge of the system's internal structure and organization. However, in most cases, such knowledge is lacking. The transfer function approach, on the other hand, treats the system as a ‘black box’, producing a non‐parametric model that eliminates subjective bias from the investigator. The trade‐off is that this model can make it difficult to extract information about the functions of individual subsystems.

Figure 1 shows a typical transfer function from anaesthetized normotensive Sprague–Dawley rats. Similar transfer functions have been obtained in other rat strains and in conscious and anaesthetized dogs (Cupples et al., 1996; Just, 2007; Marsh et al., 1990). Typically, autoregulation occurs at frequencies below 100 mHz, with two resonance peaks – one at 100–200 mHz and another at 30–40 Hz. A major challenge has been interpreting the physiological significance of the transfer functions. Although there is general consensus on the characteristics of the transfer function, different research groups have drawn varying conclusions about the mechanisms responsible for renal blood flow autoregulation (Başar & Weiss, 1970; Başar, Ruedas et al., 1968; Sakai, Hallman et al., 1986). This discrepancy arises, in part, from the difficulty of assigning specific mechanisms – such as the myogenic response and TGF – to the distinct features of the obtained transfer functions.

**FIGURE 1:**
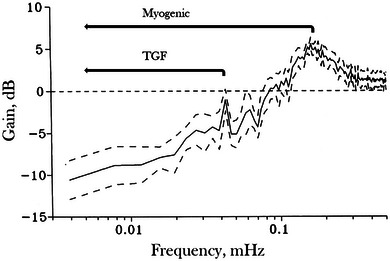
Typical transfer function showing gain in dB as a function of forcing frequency. Horizontal lines indicate the operating ranges of TGF and the myogenic mechanism. The continuous line is the mean and dashed lines represent SD. Modified from Holstein‐Rathlou et al. (1991).

To better interpret the experimental results, we pioneered the use of dynamic computational models in transfer function analysis (Holstein‐Rathlou & Marsh, 1994a; Holstein‐Rathlou et al., 1991; Sakai, Craig et al., 1986). The development of computational models for renal blood flow regulation was initially motivated by observations of spontaneous oscillations in proximal tubular pressure in anaesthetized rats (Holstein‐Rathlou & Leyssac, 1985; Holstein‐Rathlou & Marsh, 1989; Leyssac & Baumbach, 1983). These oscillations, which had a frequency of 30–40 mHz, were attributed to TGF (Holstein‐Rathlou & Marsh, 1994b; Leyssac & Baumbach, 1983). This discovery led to increased interest in TGF dynamics, prompting the development of computational models of nephron haemodynamics. Using a model incorporating dynamic representations of both the myogenic response and TGF mechanism we were able to obtain transfer functions like those observed experimentally (Holstein‐Rathlou & Marsh, 1994a).

The simulations showed that the myogenic mechanism was responsible for the resonance peak at 100–200 mHz and was the primary contributor to autoregulation above ∼40 mHz. In contrast, TGF operated at a slower pace, producing the resonance peak at 30–40 mHz and contributed mainly to autoregulation at frequences below ∼40 mHz. These findings were later confirmed by experiments where a blocker of TGF (furosemide) was shown to only affect the low frequency part of the transfer function (Just et al., 1998).

Today, there is a consensus that both the myogenic mechanism and TGF contribute to renal autoregulation (Carlström et al., 2015). The myogenic mechanism is intrinsic to preglomerular vessels and is the faster of the two, with a characteristic response time of approximately 5–10 s. In contrast, the TGF mechanism operates more slowly, with a typical response time of 20–30 s, since it relies on the transmission through the tubule of an increase in GFR to a change in [NaCl] in the tubular fluid at the macula densa (Holstein‐Rathlou & Marsh, 1994b).

An often‐overlooked issue in studies of dynamic autoregulation using spontaneous variations in arterial blood pressure is the potential bias that may arise due to covariation between system input and system response. The methods used for the analysis of dynamic autoregulation assume that the change in organ resistance (system output) is caused by the change in the perfusion pressure (system input). If this is not the case, the estimates may be biased. For example, changes in sympathetic nervous activity may simultaneously alter blood pressure and organ resistance through a direct vasoconstrictor effect on the resistance vessels throughout the body. In this case, the change in organ resistance is not a result of autoregulation but rather a consequence of altered neural activity in the organ. This could introduce systematic bias in the assessment of dynamic autoregulation. Such bias is avoided in experiments that employ external forcing of blood pressure combined with renal denervation (Holstein‐Rathlou et al., 1991; Sakai, Hallman et al., 1986).

The majority of the early work on dynamic autoregulation was on the kidneys (Başar, Ruedas et al., 1968; Holstein‐Rathlou et al., 1991; Sakai, Hallman et al., 1986), but in later years there has also been a great interest in applying the technique to the study of dynamic cerebral autoregulation (Aaslid et al., 1989; van Beek et al., 2008; Zhang et al., 1998). The underlying theory is quite general and can be applied to all sorts of dynamic physiological systems with well‐defined inputs and outputs (Marmarelis, 1978).

Only a few studies have compared static and dynamic measures of autoregulation. Bidani et al. (2003) compared static and dynamic renal autoregulation in normal and nephrectomised rats. They found a decrease in static autoregulatory activity in nephrectomised rats compared to normal rats, whereas dynamic autoregulatory measures were similar between the groups. In contrast, Tiecks et al. (1995) showed a strong correlation between static and dynamic measures of cerebral autoregulation in anaesthetized humans. Berg et al. (2012) investigated cerebral autoregulation in conscious, healthy volunteers before and after lipopolysaccharide (LPS) infusion. Whereas static autoregulation after LPS was similar to baseline, dynamic autoregulation showed decreased gain and an increased phase difference after LPS. At present it is not possible to determine whether the discrepancies between the studies are due to species differences, anaesthetized vs. conscious subjects or inter‐organ differences. Clearly, there is a need for further studies to better understand the relation between static and dynamic measures of autoregulation.

The fact that measures of static and dynamic autoregulation may differ is not surprising, since the steady‐state approach involves changes in the mean blood pressure, whereas the dynamic approach involves variations in blood pressure around a given mean. The two approaches therefore represent different physiological situations and should be viewed as complimentary ways of assessing renal autoregulatory activity. Autoregulation is important for protecting tissues and the microcirculation against pressure‐induced damage, hyperperfusion, or hypoxia due to hypoperfusion. In the kidneys, it also stabilizes GFR. Sodium and water balance is achieved through endocrine regulation of the reabsorptive processes in the distal segments of the nephron. This is a slow process, and the stabilization of GFR prevents sodium and water loss during increases in the arterial pressure, and vice versa during hypotension, due to fluctuations in tubular flow rate.

Various pathological conditions are associated with impaired autoregulation (see Carlström et al., 2015 for a recent review). Chronic hypertension, chronic kidney disease and diabetes mellitus, either alone or occurring together in the same patient, are linked to impaired autoregulation, and this may contribute to the development of end‐organ damage characteristic of these diseases. Impaired autoregulation may be a primary cause of end‐organ damage (Bidani et al., 2003) or a consequence of a primary disease process affecting autoregulatory mechanisms, thereby exacerbating end‐organ damage (Karlsen et al., 1997).

From a pathophysiological standpoint it is relevant to ask which of the two methods is better or the most relevant for assessing autoregulatory efficiency. The answer will most likely depend on the situation. In cases where there is an acute change in mean blood pressure, for example, shock or an acute hypertensive crisis, static autoregulatory efficiency is clearly of importance for mitigating the consequences of the pressure changes.

On the other hand, in many cases there is no change in mean arterial pressure, but there are constant fluctuations in the arterial blood pressure due to everyday activities (Karlsen et al., 1997). These fluctuations do not occur at specific frequencies but show what is technically known as a 1/*f* spectrum (Holstein‐Rathlou et al., 1995; Marsh et al., 1990). The implication of this is that there will be variability covering a wide range of frequencies, and that the amplitude of these will increase the lower the frequency. In other words, there will be significant variability in the blood pressure regardless of the length of the period over which it is measured. If unfiltered the pressure fluctuations will be transmitted to the microcirculation where they could lead to vascular damage. In this case, it appears likely that dynamic autoregulatory efficiency is of importance. The system not only needs the power, but also the speed to be able to reduce the effects of the pressure fluctuations on the microcirculation.

Based on the above it seems likely that the relevance of either measure of autoregulatory efficiency will depend on the situation and the characteristics of the disease or condition. This is an important field for future studies.

In most studies of dynamic autoregulation, including the use of transfer functions, it has been an implicit assumption that the system is linear. However, like most other physiological systems, the systems responsible for autoregulation are non‐linear, as evidenced by, for example, the occurrence of self‐sustained oscillations in both TGF and the myogenic mechanism (Holstein‐Rathlou & Marsh, 1994b). Even though a system may be non‐linear, it may be justified to base the analysis on the assumption that linearity prevails. If the inputs are kept small, a linear description will often be appropriate. The problem is to determine when an input has a magnitude small enough to justify an assumption of linearity. The important point to consider is that differences in the results between studies could simply be due to different magnitudes in the inputs (the arterial pressures) used to perturb the system (Marmarelis, 1988, 1989; Marmarelis et al., 1993). Unfortunately, this is a parameter that is rarely reported in published studies.

Because of the intrinsic non‐linearities of physiological systems, there has been a great effort in developing analytical methods appropriate for such systems (Marmarelis, 1988, 1989; Marmarelis et al., 1993). Space does not permit a description of these new techniques, but for a short review of the methods see Holstein‐Rathlou & Marsh (1994b). In one study, Marmarelis et al. (1993) could show that when only the linear transfer function was used, up to 20% of the variation in renal blood flow was unexplained. When second and third order non‐linearities were included in the model, the unexplained fraction of the variability was less than 7%. This shows that even when using moderate forcings of the arterial blood pressure, non‐linearities in the system are excited, and this needs to be included in the interpretation of the obtained transfer functions.

The non‐linear techniques also allow an investigation of possible interactions between the autoregulatory mechanisms. In a study in anaesthetized rats Chon et al. (1994) showed that strong non‐linearities in the autoregulatory mechanisms were present at the frequencies ∼30 and ∼130 mHz, exactly those frequencies where autonomous oscillations have been described in the rat renal microcirculation (Holstein‐Rathlou & Marsh, 1994b). The non‐linearity at ∼30 mHz corresponds to the frequency of the TGF‐mediated oscillation, and the non‐linearity at ∼130 mHz corresponds to the frequency of vasomotion. In addition, they were able to show that the two mechanisms were interacting, that is, that the activity in one of the mechanisms influenced the activity of the other. It is interesting that these techniques can detect non‐linearities, which stem from non‐linear behaviour at the microcirculatory level, in whole kidney blood flow.

Overall, improving our understanding of the interplay between static and dynamic autoregulatory measures, as well as the impact of non‐linearity, is crucial for advancing physiological and pathological assessments of autoregulation.

## AUTHOR CONTRIBUTIONS

Niels‐Henrik Holstein‐Rathlou and Donald J. Marsh both contributed to the conception of the work. Niels‐Henrik Holstein‐Rathlou wrote the first draft, and Donald J. Marsh revised it for intellectual content. Both authors approved the final version of the manuscript and agree to be accountable for all aspects of the work in ensuring that questions related to the accuracy or integrity of any part of the work are appropriately investigated and resolved, and all persons designated as authors qualify for authorship, and all those who qualify for authorship are listed.

## CONFLICT OF INTEREST

None declared.

## FUNDING INFORMATION

No funding was received for this work.
